# Post-COVID-19 arthritis: viral arthritis, not reactive arthritis

**DOI:** 10.1016/j.ero.2025.10.008

**Published:** 2025-12-09

**Authors:** Shigeto Kobayashi, Yoshinori Taniguchi, Issei Kida, Kurisu Tada, Naoto Tamura

**Affiliations:** 1 Department of Rheumatology and Internal Medicine, Juntendo University Koshigaya Hospital, Koshigaya, Saitama, Japan; 2 Department of Rheumatology, Bay Side Misato Medical Center, Kochi, Japan; 3 Department of Endocrinology, Metabolism, Nephrology and Rheumatology, Kochi Medical School Hospital, Nankoku, Japan; 4 Department of Internal Medicine and Rheumatology, Juntendo University School of Medicine, Bunkyo, Tokyo, Japan

Dear Editor,

Arthritis following SARS-CoV-2 infection or vaccination is frequently labelled as reactive arthritis (ReA) in published case reports. However, these cases are more accurately classified as ‘viral arthritis’ based on rheumatologic and virological evidence. This misclassification suggests a persistent knowledge gap in classification and understanding.

The term ReA was introduced by Ahvonen et al [1] to describe nonsuppurative arthritis triggered by extra-articular infections in which no microorganisms are detected in synovial fluid. This concept was formalised at the 4th International Reactive Arthritis Workshop in 1999, identifying bacterial pathogens—such as *Chlamydia trachomatis, Yersinia* spp., and *Salmonella* spp.—as classical triggers, typically following gastrointestinal or urogenital infection. HIV-associated ReA is generally attributed to concurrent bacterial infection rather than the virus itself. ReA has subsequently been classified within the spondyloarthritis (SpA) spectrum based on its clinical features and association with human leukocyte antigen B27 (HLA-B27). In 2011, the Assessment of SpondyloArthritis International Society (ASAS) proposed classification criteria for peripheral SpA [2]. These criteria specify preceding gastrointestinal or urogenital infections; respiratory infections are not considered typical triggers.

Despite numerous reports of ‘post-COVID-19 ReA’, many cases do not meet ASAS criteria. Gracia-Ramos et al [3] found that only 26.1% of such cases fulfilled the SpA classification criteria. This finding underscores the need to reconsider the use of the term ‘ReA’ for SARS-CoV-2–associated arthritis.

Viral arthritis is a well-established entity, often involving immune complex deposition [4]. During viremia, viral particles can enter synovial tissue, and viral DNA, RNA, or proteins have been documented in joint fluid, especially in parvovirus B19 and rubella infections. Notably, SARS-CoV-2 binds to angiotensin-converting enzyme 2 receptors, which are upregulated in inflamed synovial tissue via IL-6/STAT3 signalling, facilitating viral persistence. Two reports have demonstrated SARS-CoV-2 RNA in synovial fluid from patients with COVID-19–related arthritis [5,6]. This evidence contradicts a core criterion of ReA [1]—namely, the absence of intra-articular microorganisms.

Clinically, COVID-19–related arthritis typically presents as mono-or oligo-articular, and occasionally polyarticular, asymmetric arthritis resembling ReA, most often affecting the knees or ankles. Sacroiliac or spinal involvement is rare. Synovial fluid is usually mildly turbid to turbid, with leukocyte counts from 2000 to 20,000 cells/μL. COVID-19–related arthritis is thought to be immune-complex–mediated arthritis rather than direct virus-mediated arthritis [4]. Anti-SARS-CoV-2 IgG antibodies are produced approximately 10 days after infection, followed by the formation of immune complexes. Accordingly, arthritis developing within 4 weeks of infection reflects immune-complex–mediated viral arthritis, rather than ReA.

The timing of onset is similar for viral arthritis and ReA, typically occurring within 4 weeks after infection. According to Gracia-Ramos et al [3], COVID-19–related arthritis begins on average 20.2 days postinfection. Likewise, arthritis due to parvovirus B19 develops 10-21 days after infection, rubella within 7-21 days, and postvaccination arthritis typically 2-6 weeks after live-virus vaccination.

However, a key distinguishing feature is symptom duration: viral arthritis usually resolves within 6 weeks [4], whereas ReA often persists beyond 3 months. In our previous analysis, the mean duration of post-COVID-19 arthritis was only 17 days (range 2-30 days), supporting its classification as transient viral arthritis rather than ReA. To clarify these distinctions, we provide a comparative summary of viral arthritis and classic ReA (Table).

**Table:** Clinical and immunological differences between viral arthritis and reactive arthritis

Feature		Viral arthritis	Reactive arthritis
**Trigger**		Viral infection (eg, parvovirus B19, rubella, SARS-CoV-2, hepatitis viruses) or vaccination	Bacterial infection (eg, Chlamydia trachomatis, Salmonella, Yersinia, Shigella, Campylobacter)
**Pathogenesis**		Direct invasion of synovium (rubella virus and parvovirus B19); Immunocomplex mediated (hepatitis B virus, hepatitis C virus, and post-COVID-19); Immune dysregulation (Epstein–Barr virus, HTLV-1)	Persistence of bacterial antigens; molecular mimicry; HLA-B27 heavy chain misfolding
**Onset**		Often coincides with or shortly follows systemic symptoms, 1-4 wk after infection. May occur after an asymptomatic viral infection	1-4 wk after infection. Often follows symptomatic infection
**Pathogenesis**		1. Direct invasion to synovial tissue(rubella virus and parvovirus B19) 2. Immunocomplex arthritis (Hepatitis B virus, Hepatitis C virus, and post-COVID-19) 3. Immune dysregulation (Epstein–Barr virus, HTLV-1)	Persistence of bacterial DNA and fragments, molecular mimicry between bacterial and host molecules (eg, HLA-B27), and HLA-B27 heavy chain misfolding
**Microbial detection in joint**		COVID-19: viral RNA/protein may be detectable in synovial fluid	No viable microorganisms or bacterial DNA detectable
**Arthritis**	**Peripheral**	COVID-19; mostly oligo -or monarthritis, large joints mainly knees and ankles, occasionally wrists and elbows	Asymmetric oligoarthritis, lower limbs
	**Sacroilliac/spinal joint**	COVID-19: very rare, isolated sacroillitis in a few MRI-based reports	Common in ReA and SpA spectrum
**Synovial fluid**		COVID-19: clear–mildly turbid; WBC 2000-20,000 cells/μL	Turbid; WBC 10,000–50,000 cells/μL
**Extra-articular manifestations**		COVID-19: ‘enthesitis, others’	Conjunctivitis, urethritis or cervicitis, oral ulcers, dactylitis, enthesitis, keratoderma blennorrhagicum, circinate balanitis
**Duration of arthritis**		Short, typically <6 wk in viral arthritis, COVID-19; mean 17 d (range: 2-30 d) (based on the authors’ analysis)	Longer, remission in 3-12 mo; ∼25% may develop chronic disease
**HLA-B27 association**		Absent. Not typically associated (<5∼10%)	Strong association (30∼90%)
**Classification criteria**		Not included in ASAS classification or 1999 definition; considered outside spondyloarthritis spectrum	Meets ASAS criteria and definitions from the 1999 4th International Reactive Arthritis Workshop

ASAS, Assessment of SpondyloArthritis International Society; HLA-B27, human leukocyte antigen B27; HTLV-1, human T cell leukemia virus type 1; IgG, immunoglobulin G; ReA, reactive arthritis; SpA, spondyloarthritis; WBC, white blood cell.

Note: COVID-19–associated arthritis is thought to result from immunocomplex formation. Antiviral IgG antibodies typically emerge 7-10 days after infection, potentially leading to the formation of immune complexes and the onset of arthritis within 4 weeks. This delayed onset may contribute to misclassification as post-COVID-19 reactive arthritis. Once immune complexes are cleared, arthritis generally resolves within 42 days, whereas ReA often persists for more than 3 months.

We urge clinicians and reviewers to adopt more precise terminology—such as *post-COVID-19 viral arthritis, COVID-19–related viral arthritis*, or *postvaccination viral arthritis*. Continued use of the term *reactive arthritis* for SARS-CoV-2–associated joint manifestations reflects an incomplete understanding of postviral immunopathology compared with classical spondyloarthritis. A mechanism- and definition-based classification will improve diagnostic precision, patient care, and scholarly communication.

## CRediT authorship contribution statement

**Shigeto Kobayashi:** Writing – review & editing, Writing – original draft, Project administration, Investigation, Formal analysis, Data curation, Conceptualization. **Yoshinori Taniguchi:** Writing – review & editing, Conceptualization. **Issei Kida:** Writing – review & editing, Resources, Investigation, Data curation. **Kurisu Tada:** Writing – review & editing, Writing – original draft, Investigation, Data curation. **Naoto Tamura:** Writing – review & editing, Supervision, Project administration, Formal analysis, Conceptualization.
